# SARS-CoV-2 nucleocapsid protein undergoes liquid–liquid phase separation into stress granules through its N-terminal intrinsically disordered region

**DOI:** 10.1038/s41421-020-00240-3

**Published:** 2021-01-21

**Authors:** Jia Wang, Chengrui Shi, Qun Xu, Hang Yin

**Affiliations:** 1 School of Pharmaceutical Sciences, Tsinghua University, Beijing 100084, China; 2 Tsinghua-Peking Center for Life Sciences, Tsinghua University, Beijing 100084, China; 3 Beijing Advanced Innovation Center for Structural Biology, Tsinghua University, Beijing 100084, China; 4 Department of Chemistry, Tsinghua University, Beijing 100084, China

**Keywords:** Immunology, Cell signalling

Dear Editor,

The novel coronavirus disease 19 (COVID-19) broke out in late 2019 and has spread worldwide since then, posing a great threat to the world economy and public health. As of December 1, 2020, more than 62.8 million patients have been diagnosed worldwide, causing 1.46 million deaths with a mortality rate of more than 2.3% (source: WHO situation report), possibly making it one of the deadliest pandemics in the entire human history. Unfortunately, the clinical efficacy of a number of potential drugs remains to be vague. Therefore, further in-depth research on the physiological processes of virus transmission, infection, replication, release, and the identification of novel therapeutic targets are of remarkable significance for the prevention and treatment of COVID-19.

The causative agent of COVID-19, SARS-CoV-2, is a positive-sense, single-stranded RNA virus with a 30 kb genome that has 80% sequence homology with SARS-CoV, transcribing up to 29 proteins^1^. The multivalent, RNA-binding nucleocapsid (N) protein stabilizes the genomic RNA inside the virus particle. Furthermore, the N protein regulates essential processes, including the viral genome transcription, replication and packaging. The N protein can be divided into several domains from the N-terminus to the C-terminus: the N-arm; the N-terminal domain (NTD), which can bind to the genome RNA; the linker region; the C-terminal domain (CTD), which is responsible for N protein dimerization in a form of CTD–CTD interaction and also binds RNA; and the C-tail^2^. X-ray crystal structures of the NTD and the CTD of N protein have been solved^3–5^. The higher-order structure of ribonucleoproteins at 13.1 Å has revealed an intriguing reverse G-shape^6^. However, it remains obscure how N protein interferes with physical activities in cell during the whole virus replication cycle.

Liquid–liquid phase separation (LLPS) is a process by which biomolecules, such as proteins or nucleic acids, condense into a dense phase that often resembles liquid droplets^7^. This dense phase could coexist with a dilute phase. Molecules of phase-separated droplets always rapidly exchange with the soluble phase and behave macroscopically as liquids, while sometimes these droplets mature into solids. During virus infection, LLPS serves as a scaffold for virus replication and promotes the assembly of viral machinery for virus production through proximity-dependent interactions^8^.

Cytoplasmic stress granules (SGs) are composed of messenger RNAs (mRNAs), translation initiation factors, mRNA-binding proteins that provide translational control, mRNA stability proteins related to mRNA metabolism and some signaling proteins^9^. Upon cellular stress, SGs are induced to trigger global translational silencing. Coronaviruses such as mouse hepatitis coronavirus and transmissible gastroenteritis virus were reported to induce SG assembly^10^. The SARS-CoV N protein could translocate to cytoplasmic SGs, while this process is inhibited by phosphorylation of its serine/arginine (SR) motif^11^. In a recent paper, an affinity-purification mass spectrometry analysis showed that SARS-CoV-2 N protein binds to the SG proteins such as GTPase-activating protein-binding proteins 1 and 2 (G3BP1/2) as well as other host mRNA-binding proteins and mRNA decay factors^12^. Nevertheless, whether SARS-CoV-2 N protein condenses into SGs was previously unknown.

In this study, we report that the SARS-CoV-2 N protein undergoes LLPS through its N-terminal intrinsically disordered region (IDR) with G3BP1 into SGs. To examine the capacity of N protein to phase separate, we first analyzed the N protein sequence using the PLAAC (prion-like amino acid composition) tool. The results showed that the N terminal 1–39 amino acid (aa) region was an IDR, which was enriched with disorder-promoting residues such as proline, glycine, serine, alanine, and asparagine (Fig. 1a). Since plenty of IDRs can lead to LLPS, we tested whether N protein can form phase condensates. In HeLa cells, overexpressed GFP-tagged N protein was diffused in the cytosol. To model the stressed state of cells infected with SARS-CoV-2, we added arsenite into the culture medium. Interestingly, upon arsenite stimulation, we observed that N protein formed droplets of several micrometers, suggesting occurrence of their condensation (Fig. 1b). Moreover, we also asked whether N-terminal IDR was important for N protein condensation by overexpressing ∆N (N protein without N-terminal 1–39 aa region) truncation in HeLa cells. Subsequent studies showed that removal of N-terminal IDR impaired protein aggregation and droplet formation (Fig. 1b). Statistical analyses showed that without N-terminal IDR, the percentage of cells with puncta dropped by a half (Supplementary Fig. S1a, b). This result indicates that N-terminal IDR is necessary for SARS-CoV-2 N protein aggregation.

**Fig. 1:**
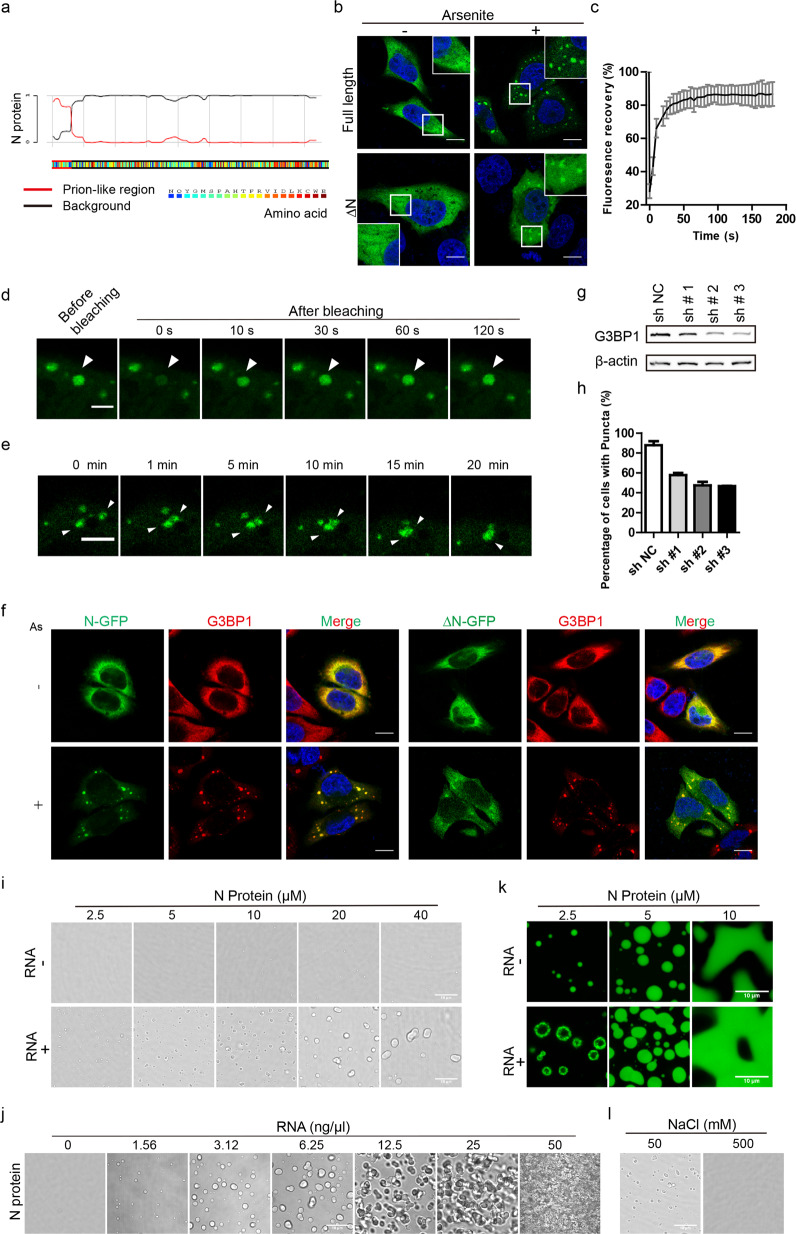
SARS-CoV-2 N protein phase separates into stress granules in cells with G3BP1 and in vitro. **a** Prion-like domain analysis of SARS-CoV-2 N protein revealed that the N-terminal 1–39 aa was a probable IDR, which inclined to undergo LLPS. **b** Images of HeLa cells overexpressing GFP-tagged N protein (top) or N protein without the N-terminal 1–39 residues (∆N) (bottom). The left and right panels showed cells in the absence and presence of arsenite at 500 μM for 30 min, respectively. N protein aggregated into spherical droplets of several micrometers in diameter. Scale bar, 10 μm. **c**, **d** Fluorescence recovery of N-GFP protein after photobleaching showed that N protein demonstrated exceptional recovery in 60 s. Scale bar, 5 μm. The error bar represents SD (*n* = 18). **e** N-GFP protein underwent droplet fusion in a time-lapse movie after arsenite stress. Scale bar, 5 μm. **f** Fluorescence images of N-GFP condensation with endogenous G3BP1. ∆N-GFP failed to phase separate into G3BP1 SGs. Arsenite was added into medium at 500 μΜ for 30 min. Scale bar, 10 μm. As, arsenite. **g** Western blotting of HeLa G3BP1 knockdown cell lines showed effective knockdown by #2 and #3 shRNAs. **h** Statistic analysis showing that HeLa cells formed N-GFP puncta after G3BP1 knockdown with arsenite treatment for 30 min at 500 μM. For each group of cells, more than 50 cells were calculated for each time. **i** Upper panel, SARS-CoV-2 N proteins were diluted at 2.5, 5, 10, 20, and 40 μM separately in 25 mM Hepes (pH 7.5), 50 mM NaCl, and observed under bright field of a confocal microscope. Lower panel, SARS-CoV-2 N protein were diluted at 2.5, 5, 10, 20, and 40 μM separately in 25 mM Hepes (pH 7.5), 50 mM NaCl with additional 6.25 ng/μL total RNAs from HeLa cells. Droplets were observed under a bright field of a confocal microscope. Scale bar, 10 μm. **j** N protein at 10 μM in 25 mM Hepes (pH 7.5), 50 mM NaCl were stimulated with increased concentration of total RNAs. Scale bar, 10 μm. **k** FITC-labeled N protein were observed under 488 laser at different concentrations with or without RNA stimulation. The concentration of added HeLa cell total RNAs was 25 ng/μL. Scale bar, 10 μm. **l** Left, N proteins at 10 μM were stimulated with 6.25 ng/μL HeLa cell total RNAs in 25 mM Hepes (pH 7.5), 50 mM NaCl; right, the salt concentration of the left protein solution was raised to 500 mM and droplets dissolved. Scale bar, 10 μm.

Next, we evaluated whether N protein possesses other characteristics of phase separation proteins. A quantitative assessment of N protein mobility using fluorescence recovery after photobleaching in live cells showed that N protein fluorescence signals recovered quickly after bleaching. Specifically, 50% of the original fluorescence signals recovered in 5–10 s after photobleaching (Fig. 1c, d), suggesting a high fluidity of the puncta and a rapid exchange rate of the puncta with the cytoplasm. Live cell imaging of puncta under arsenite stimulation exhibited that two small droplets moved into proximity with each other, contacted and finally fused into a larger droplet over the course of 20 min (Fig. 1e), representing the fusion feature of LLPS. Taken together, these observations demonstrate that N protein exhibits charateristics of LLPS in live cells under stress.

Furthermore, the percentage of puncta-containing cells increased with arsenite induction over time, suggesting that the process was dependent on proteins in SGs (Supplementary Fig. S1c). The core components of SGs are G3BP1 and G3BP2, without which the SGs would fail to form in cells. To learn whether G3BP1 was involved in the N protein condensation, we conducted immunostaining of endogenous G3BP1 in unstressed and stressed HeLa cells, which displayed well co-localization of G3BP1 with N protein puncta under arsenite induction (Fig. 1f). However, the ∆N truncation was mostly diffused in cells (Fig. 1f). In addition, we overexpressed N-GFP or ∆N-GFP with mCherry-G3BP1 fusion protein in HeLa cells. Without arsenite stimulation, they were distributed dispersedly in the cytoplasm. However, upon arsenite stimulation, most N-GFP protein formed condensates together with mCherry-G3BP1 (Supplementary Fig. S1d). As for ∆N-GFP, droplets were only observed in partial cells, while most ∆N-GFP and mCherry-G3BP1 proteins stayed diffused in the cytosol (Supplementary Fig. S1d). These data indicate that N protein condensates are related to G3BP1 and the N-terminal IDR of N protein contributes to its localization. To explore whether the condensate development is dependent on endogenous G3BP1, we established G3BP1 stable knockdown HeLa cell lines with three different shRNAs, which all effectively reduced G3BP1 protein level (Fig. 1g; Supplementary Fig. S1e). Overexpressing N-GFP in these cells, we found that depletion of G3BP1 suppressed N-GFP puncta formation after arsenite treatment (Fig. 1h; Supplementary Fig. S1f), indicating that condensation of N protein into SGs relies on G3BP1 protein.

To rule out the influence of non-specific factors in HeLa cells, we set out to reconstitute N protein condensates in vitro. We expressed GST-tagged N protein in *Escherichia coli*, purified the GST-N protein, removed the GST tag, and then loaded the protein through a Superdex 200 gel filtration column. The purity of the protein was verified by SDS-PAGE analysis (Supplementary Fig. S2a, b). Next, we diluted the protein in a physiological buffer at different concentrations and observed no droplets below 10 μΜ (Fig. 1i). When the protein concentration reached 10 μΜ, droplets smaller than 1 μm appeared in the solution, but the droplet sizes did not grow even at a protein concentration of 40 μΜ (Fig. 1i), indicating extra factors are needed for N protein condensates in vitro. Considering the ability of N protein to bind RNAs, we added total RNAs extracted from HeLa cells into N protein solutions. Droplets appeared with the supplement of RNAs (Fig. 1i). Moreover, the size of condensates increased with higher protein concentration meanwhile their numbers decreased (Fig. 1i). Next, with protein concentration fixed at 10 μΜ along with an increasing RNA gradient, we observed that N protein condensed into droplets, then gel-like structure and finally solid-like entanglement (Fig. 1j), suggesting RNAs play a crucial role in this process. We also labeled purified N protein with FITC and observed its assembly at different protein concentrations in the presence and absence of RNAs (Fig. 1k). These results were similar to those obtained with unlabeled protein, showing dependence on the concentration of protein and the addition of RNAs (Fig. 1i). Recently it was reported that G3BP1–RNA interaction drove the assembly of SGs^13^. Since both N protein and G3BP1 interact with RNAs, the sequential steps of N protein phase separation into SGs remain to be an important question. Moreover, increased salt concentration led to the dissolution of droplets (Fig. 1l), suggesting that the process is reversible. Taken together, our data demonstrate that the SARS-CoV-2 N protein phase separates into droplet-like structures in cells and in vitro. Furthermore, this process is regulated by G3BP1 protein and by RNAs.

We next investigated the contributions of different N protein domains to N protein LLPS. Various N protein truncations and deletions were constructed and transfected into HeLa cells, which were then treated or not with arsenite. The results revealed that cells expressing the 1–174 fragment (N-terminal IDR plus NTD) formed puncta even without stimulation, while NTD or CTD alone could barely form puncta even with arsenite addition (Supplementary Fig. S3a, c). These data suggest that the N-terminal IDR is indispensable for the N protein condensation. By comparison, full-length N protein formed puncta only after stimulation, however, cells expressing the 1–174 aa fragment formed puncta spontaneously (Supplementary Fig. S3a, c). This result suggests that 175–419 aa region might act as an auto-inhibitory domain in N protein LLPS. Meanwhile, cells expressing constructs without the SR motif (∆176–206, ∆176–246) had more puncta in cells (Supplementary Fig. S3a, c), suggesting that phosphorylation may inhibit N protein LLPS. After ensuring comparable protein expression levels of the different constructs (Supplementary Fig. S3b), a comparison of the number and area of puncta per cell demonstrated no significant differences between the full-length N protein and the 1–174 truncation (Supplementary Fig. S3d, e). By contrast, the SR motif deletion remarkably augmented both the number and area of puncta (Supplementary Fig. S3d, e).

Finally, we would like to point out that while we were in the process of preparing this manuscript, a paper was published showing that N protein phase separated with RNA and that this process could be enhanced by Zn^2+^^14^. Similar to these findings, we also demonstrated that N protein phase separation was triggered by HeLa cell total RNAs in a dose-dependent manner. Furthermore, our data prove N protein phase separation both in cell and in vitro. Results from the in vitro experiments exhibited that HeLa cell RNAs alone can stimulate the formation of N protein condensates, thus, the precise role of G3BP1 needs further investigation. Since viruses rely on host cells to synthesize proteins, the formation of SGs and subsequent translation arrest serve as a universal antiviral response^15^. The condensation of N protein into SGs could be a strategy of SARS-CoV-2 inhibiting host cell innate immunity. In summary, our findings reveal a critical domain responsible for N protein phase separation and the dynamic organization of N protein into SGs in cells. Disruption of N protein LLPS holds promise for antiviral intervention. The special interaction between N protein and G3BP1 offered new targets and strategies for the development of drugs to combat COVID-19.

## Supplementary information

supplemental material

## Supplementary information

**Supplementary Information** accompanies the paper at (10.1038/s41421-020-00240-3).
