# Supplementary material for: SARS-CoV-2 nucleocapsid protein undergoes liquid–liquid phase separation into stress granules through its N-terminal intrinsically disordered region
Source: Cell Discov. 2021 Jan 21;7:5. doi: 10.1038/s41421-020-00240-3 (PMC7817956; doi:10.1038/s41421-020-00240-3)
Supplement: Supplementary file 1 — supplemental material [file 41421_2020_240_MOESM1_ESM.pdf]

Supplementary information

Materials and Methods

Plasmid constructs

The cDNA encoding N protein was cloned into pEGFPN1 plasmid using the standard polymerase chain reaction (PCR) and restriction enzymes method. Truncations and deletions of N plasmid were made using similar method with appropriate primers. DNA fragments encoding N protein were inserted into pGEX-6P-1 plasmid using pEASY-Uni Seamless Cloning and Assembly Kit (Transgen, CU101) for purification of N protein. The pEGFP-C1-G3BP1 plasmid (#135997) was purchased from Addgene. DNA fragments encoding mCherry and G3BP1 were amplified using mCherry-N1 plasmid and pEGFP-C1-G3BP1 plasmid separately, then they were assembled and inserted into pCDNA3.1(-) plasmid by pEASY-Uni Seamless Cloning and Assembly Kit.

Cell culture

HeLa cells were cultured in DMEM supplemented with 10% FBS, 2 mM L-glutamine, 100 μg/ml streptomycin, and 100 U/ml penicillin. Cells were maintained at 37°C in an incubator with 5% CO_2. The plasmids were transfected into HeLa cells using lipofectamine 3000 reagents (Thermo Fisher) according to the manufacturer's instructions.

Fluorescence imaging

Cells were transfected with indicated plasmids and grown on coverslips for 20-24 h. After arsenite treatment at indicated time points, cells were washed twice with PBS, fixed with 4% paraformaldehyde, followed by incubation with DAPI (0.1μg/ml) (Sigma Aldrich, #D9542) in PBS and sealed with mounting buffer (Sigma Aldrich, #F4680). For immunostaining, after fixation, cells were incubated with 5% BSA, 0.1% Trition X-100 in PBS for 45 min, then washed three times with PBS, followed by incubation with G3BP1 antibody (CST, #61559) diluted in incubation buffer (1% BSA, 0.02% Triton X-100 in PBS) for one hour at room temperature. After wash three times with PBS, the samples were incubated with Alexa Fluor 568 conjugated secondary antibody (Thermo Fisher, #A11011) for one hour respectively, then stained with DAPI (0.1μg/ml) in PBS and sealed with mounting buffer. Images were collected using a Nikon A1R MP confocal microscope under an 100× oil-immersion objective. All images were processed with Image J software.

Western blotting

The HeLa cells were transfected with indicated plasmids. 24 hours post transfection, after arsenite stimulation, the cells were collected, lysed with 1 x protein loading buffer and subjected to SDS-PAGE. The antibodies used were as follows: GFP (Abclonal, #AE012), β-actin (CST,#3700S), G3BP1 (CST, #61559).

FRAP and live cell imaging

HeLa Cells were transfected with indicated plasmids and grown on a culture dish with glass bottom for 20-24 h. Cells were transferred to a living cell station (Oko Lab) and cultured at 37°C, 5% CO_2, and 80% humidity. Arsenite was added 30 min before observation under Nikon A1R MP confocal microscope. For FRAP experiment, the green puncta of

appropriate size were chosen and bleached by 0.25 s with 100% laser power (488 solid state laser), whereafter scanned with a 30 s interval of 3 min in total. Mean optical intensity of the bleached puncta were calculated applying Image J software. All collected data were analyzed with Excel 2013 software and graphed in Graphpad Prism 5 software. For live cell imaging, the cells with droplets were photographed at 30 s interval in a duration of 1 hour. All images were processed with Image J software.

G3BP1 stable knock down cell lines

shRNA plasmids (#1, TRCN0000008721; #2, TRCN0000008720; #3, TRCN0000008722) for knocking down of human G3BP1 were from Sigma Aldrich. 293T/17 cells were transfected with the indicated shRNA plasmids together with psPAX2 and pMD2.G plasmids using lipofactamine 3000 reagents. 18 hours after transfection, the supernatant were replaced with DMEM with 10%FBS. The virus were collected at 48, 72 hours post transfection. The pooled viruses were mixed with polybryne (10 μg/ml) and supplied to HeLa cells. 24 hours post viruses infection, HeLa cells were supplemented with fresh medium containing puromycin (3 μg/ml) for 48 hours to select cells successfully inserted with shRNA.

Statistics of puncta

The criteria we used to define the ratio of cells with puncta were as follows: The number of N-GFP expressing cells bearing more than one puncta with a diameter of more than one micrometer was calculated as *a*. The number of N-GFP expressing cells without puncta was calculated as *b*. The percentage of cells with puncta equaled *a/(a+b)*.

The number and area of puncta in cells were automatically calculated using ImageJ software and analyzed by Excel software.

Protein purification

BL21 (DE3) competent *E.*coli (TIANGEN, CB105) cells were transformed with N-pGEX-6P-1 plasmids. Single colonies were grown at 37 ℃ in LB media containing 50 μg/ml Ampicillins. *E.*coli cells were grown to OD_{600} of 0.8 and induced with 0.5 mM IPTG at 18 ℃ for 16 hours. Cells were collected by centrifugation, suspended in lysis buffer (25 mM HEPES, 1M NaCl, pH 7.5) supplemented with 1 mM PMSF, 0.1mg/ml RNase A and disrupted by high pressure. Lysates were centrifuged at 12,000 rpm at 4 ℃ for 1 hour. The supernatant were loaded onto columns packed with GST beads (GE, 170756). GST-N proteins were eluted with elution buffer (25 mM Tris, 300 mM NaCl, pH 8.0, 20 mM glutathione). The eluted proteins were cleaved by HRV 3C protease (Takara, 7360) at 4 ℃ with RNase A overnight. The cleaved proteins were further purified by a GST column and a Superdex 200 10/30 increase column (GE) equilibrated in SEC buffer (25 mM HEPES, 500 mM NaCl, pH 7.5). The fractions were checked by SDS-PAGE, flash frozen in liquid nitrogen and stored at -80 ℃.

Protein fluorescent labeling

2mg of N protein were labeled with FITC using HOOK™ Dye Labeling Kit (G-Biosciences, #786-141) according to the manufacturer’s instructions. The labeled proteins were

concentrated and flash frozen with liquid nitrogen and stored at -80 ℃.

Liquid-liquid phase separation

The in vitro LLPS experiments were conducted at room temperature. Proteins were mixed at a desired concentration, and the concentration of NaCl was adjusted with 5 M NaCl and 25 mM HEPES (pH 7.5). HeLa cell total RNAs were extracted with RNAsimple Total RNA Kit (TIANGEN, DP419). The concentration of RNAs were quantified by Nanodrop (Thermo Fisher). Protein solutions were prepared 15-30 min before imaging at room temperature.For imaging, protein solutions were loaded onto a glass-bottom 384 well plate. Images were taken by Nikon A1R MP confocal microscope using a 100× oil-immersion objective.

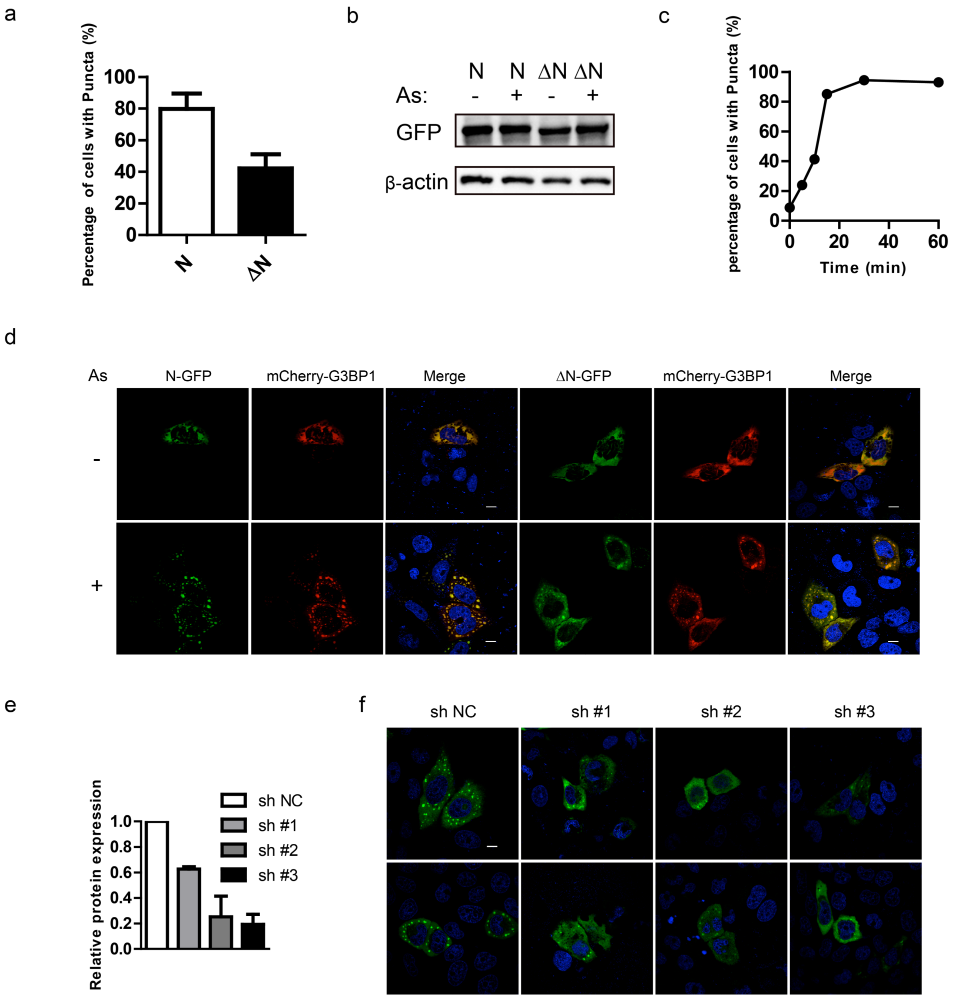

Supplementary information, fig. S1 N protein condensation relies on N-IDR and G3BP1. a, Statistics of puncta in cells overexpressing N-GFP and ΔN-GFP. **b**, Protein expression level of N-GFP and ΔN-GFP in HeLa cells. **c**, Percentage change of HeLa cells with N-GFP puncta in a time course treatment of arsenite at 500 μM. ***, p< 0.001. **d**, left panel: Fluorescence images of N-GFP condensation with overexpressed mCherry-G3BP1. right panel: ΔN-GFP failed to phase separate into mCherry-G3BP1 SGs. Arsenite were added into the medium at 500 μM for 30min. Scale bar, 10μm. **e**, Statistic analysis of G3BP1 protein knock down in HeLa cells. #No. 2 and #No. 3 shRNAs showed about 80% knockdown of G3BP1. The results were average of three independent replicates. **f**, Represent images of N-GFP puncta after arsenite stimulation at 500 μM for 30 min in three G3BP1 knockdown cells respectively. Scale bar, 10μm. As: arsenite.

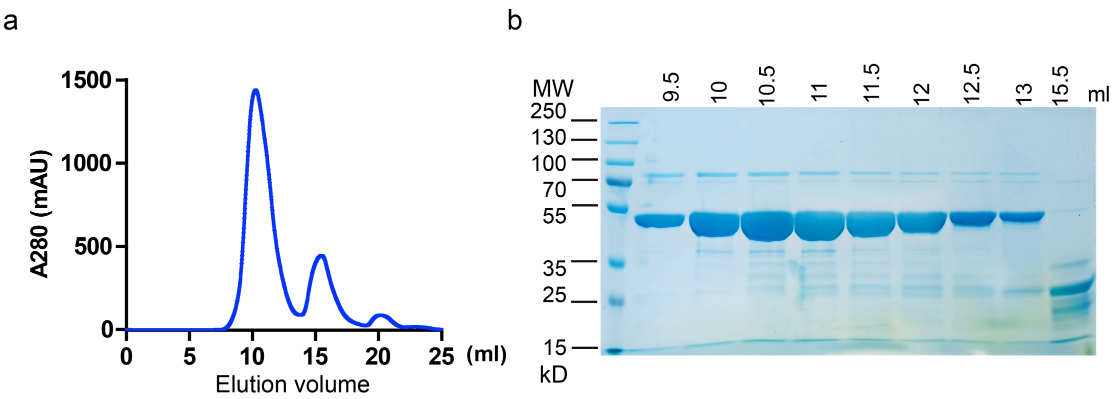

Supplementary information, fig. S2 Purification of N protein. a, Superdex 200 gel filtration result of purified N protein. **b**, The fractions obtained from Superdex 200 gel filtration were analyzed by SDS-PAGE.

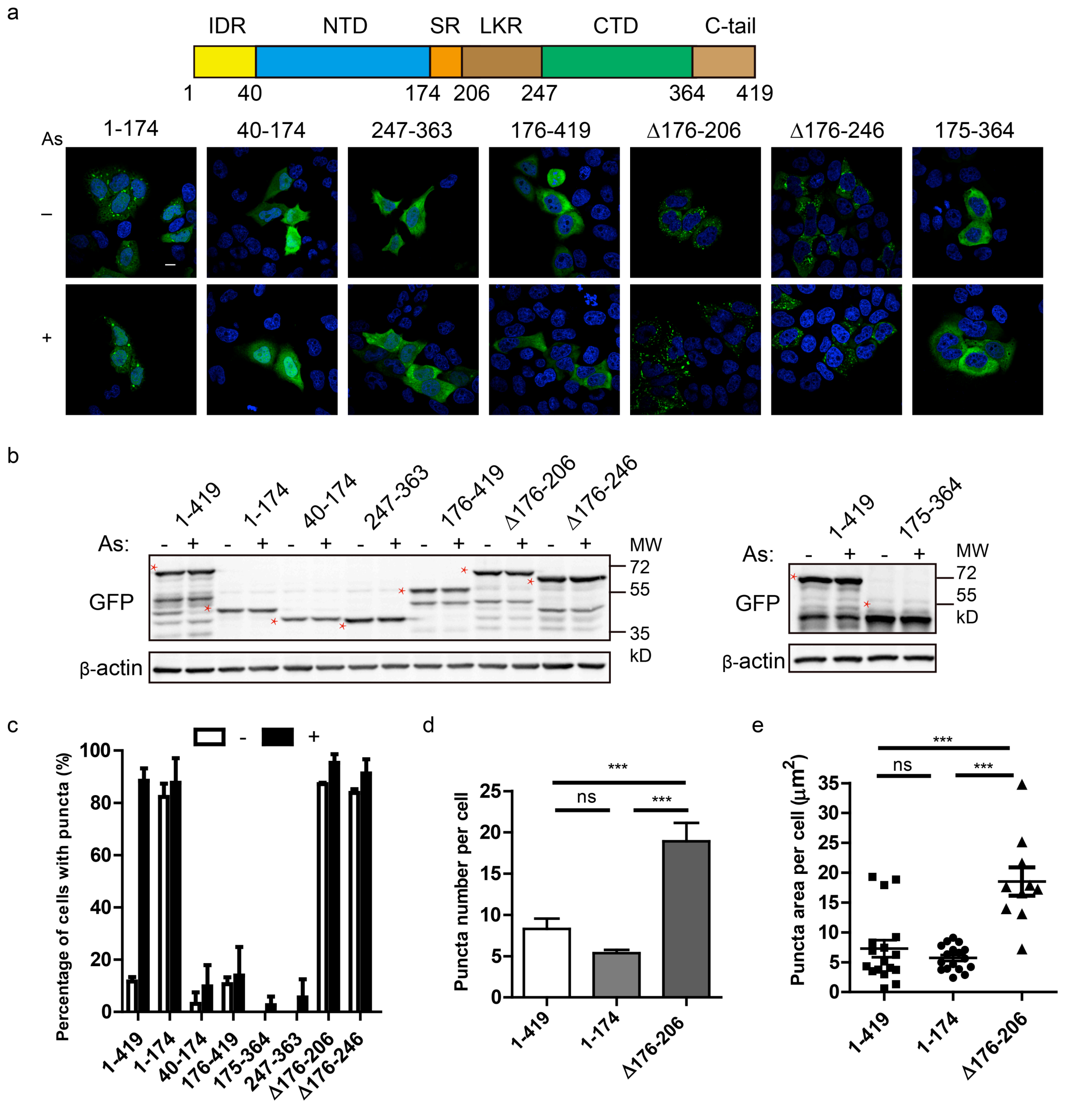

Supplementary information, fig. S3 Dissecting of different domains in N protein phase separation. a, Images of HeLa cells overexpressing the corresponding truncations and deletions in the absence and in the presence of arsenite at 500 μM for 30 min. Scale bar, 10μm. **b**, Protein expression level of the corresponding constructs in HeLa cells. **c**, Statistics of percentage of cells with puncta. **d**, Statistics of the number of puncta per cell in the absence and in the presence of arsenite. More than 30 cells were calculated in each group. **e**, Statistics of the total area of puncta per cell. More than 30 cells were calculated in each group. As: arsenite.
